# Label-free superior contrast with c-band ultra-violet extinction microscopy

**DOI:** 10.1038/s41377-023-01105-6

**Published:** 2023-03-03

**Authors:** Florian Ströhl, Deanna L. Wolfson, Ida S. Opstad, Daniel H. Hansen, Hong Mao, Balpreet S. Ahluwalia

**Affiliations:** 1 Department of Physics and Technology, UiT The Arctic University of Norway, Tromsø, Norway; 2 Department of Clinical Science, Intervention and Technology, Karolinska Institute, Stockholm, Sweden

**Keywords:** Microscopy, Biophotonics, Optical sensors

## Abstract

In 1934, Frits Zernike demonstrated that it is possible to exploit the sample’s refractive index to obtain superior contrast images of biological cells. The refractive index contrast of a cell surrounded by media yields a change in the phase and intensity of the transmitted light wave. This change can be due to either scattering or absorption caused by the sample. Most cells are transparent at visible wavelengths, which means the imaginary component of their complex refractive index, also known as extinction coefficient *k*, is close to zero. Here, we explore the use of c-band ultra-violet (UVC) light for high-contrast high-resolution label-free microscopy, as k is naturally substantially higher in the UVC than at visible wavelengths. Using differential phase contrast illumination and associated processing, we achieve a 7- to 300-fold improvement in contrast compared to visible-wavelength and UVA differential interference contrast microscopy or holotomography, and quantify the extinction coefficient distribution within liver sinusoidal endothelial cells. With a resolution down to 215 nm, we are, for the first time in a far-field label-free method, able to image individual fenestrations within their sieve plates which normally requires electron or fluorescence superresolution microscopy. UVC illumination also matches the excitation peak of intrinsically fluorescent proteins and amino acids and thus allows us to utilize autofluorescence as an independent imaging modality on the same setup.

## Introduction

High-resolution brightfield microscopy of cells is inherently limited by low image contrast. Thus, small structures inside cells that are close to but above the resolution limit are often not detected. Extrinsic stains, either absorbent or fluorescent, provide excellent contrast but require time and effort and can give varying results based on staining batch and operator; they also can alter the natural function of the cell. Intrinsic contrast methods are thus preferable.

Light passing through cells with higher refractive indices is slowed in comparison to light passing through a lower index medium, resulting in a phase difference^1^. This difference is hardly noticeable in brightfield microscopy but can be converted into visible intensity differences using techniques like phase contrast or differential interference contrast microscopy (DIC)^2^. Both techniques have been extremely successful in visualizing thin biological specimens but share a main drawback: they do not provide quantitative measurements. A camera’s recorded intensity values provide only qualitative insights and absolute pixel values do not directly correspond to physical quantities.

Label-free quantitative measurements require more advanced methods that often involve the recording of interferograms^1^. Examples include holotomography^3^, optical coherence tomography^4^, or quantitative phase microscopy (QPM)^1^, which permit the calculation of the illumination light’s optical path difference (often simply called “phase”) or the refractive index distribution of the sample. The hallmark of these techniques is their reliance on a reference beam to map minuscule differences to the probing beam via interferometry, which complicates the required optical setups. Computational QPM methods offer phase recovery without the need for interferograms but at the expense of multiple intensity measurements^5–7^. Intriguingly, QPM provides access to both the real and imaginary parts of the phase. Commonly in bioimaging, the imaginary phase information is inaccessible to users. This is because the extinction coefficient (effectively the absorption of light by the sample), which is used to extract the imaginary phase, contributes almost nothing to the image contrast. This holds for the visible light region but changes at shorter wavelengths.

**Fig. 1:**
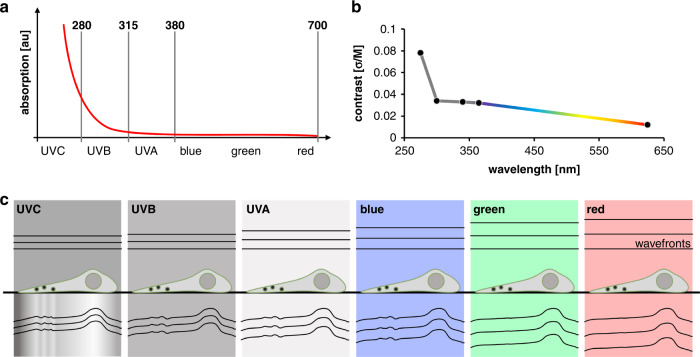
Absorption and contrast difference at different wavelengths, measured on liver cells. **a** Conceptual “hockey stick” figure of tissue absorption as observed in many biological materials^10–13^. **b** Measured contrast (standard deviation over median) at different wavelengths in brightfield microscopy of liver cells. **c** Schematic diagram of light interacting with liver cells containing nano-sized fenestrations present on the cell membrane (shown in dark circles). In the UVC, both the real refractive index and the extinction coefficient leave an appreciable imprint on the probing light. A phase retrieval algorithm can disentangle the real and imaginary parts from intensity images in a quantitative manner^14^

C-band ultra-violet (UVC) light, which spans from 200 to 280 nm, experiences much greater absorption and specificity in biological matter than visible light^8^ and offers an inherent sectioning effect due to a much lower penetration depth^9^. Biological materials that contain proteins, lipids, or nucleic acids show a distinctive “hockey stick” figure when plotting wavelength versus absorption^10–12^. Absorption increases as wavelength decreases, with a pronounced increase in the UVC (see Fig. 1a for a conceptual plot). UVC absorption is largely due to nucleic acids and proteins with peaks at 260 nm and 280 nm respectively, whereas UV-B light around 300 nm does not directly correspond to any specific cellular constituent^8^. Therefore, while the exact start of the “kink” in absorption is sample-specific and depends on the particular mix of proteins and nucleic acids in the region of interest, it is generally expected to always be located near the onset region of the UVC. From this, we can assume that the higher absorption of UVC will be mirrored in terms of higher image contrast in brightfield microscopy. This provides the intriguing possibility of using UVC light absorption as an avenue to retrieve highly contrasted, quantitative extinction coefficient maps as a complementary measurement for quantitative phase maps.

Previous work on quantitative microscopy methods in the UV predominantly used the near UV, and that was for the sole purpose to increase resolution. For instance, the work by Cotte et al.^3^ employed off-axis holographic tomography at a wavelength of 405 nm to reach a resolution below 200 nm. In the UVC, previous work mainly exploited the autofluorescence property of tissue or common dye stains as a means to improve contrast^9^ or used the selective absorption of UVC light in transmission to gain complementary sample information^8^. Previous examples of quantitative phase imaging in the UVC used either an interferometric setup^15^ or the transport of intensity equation^16^; because both approaches relied on (quasi-) coherent illumination they were thus limited to only half the maximum resolution obtainable using incoherent light. Furthermore, both were impacted by the lack of available aberration-corrected optics in the UVC, which can significantly reduce image quality. An additional limiting factor was strong chromatic aberrations in the deep UV, which are much more severe than in the visible. For instance, even the light from a typical narrow-bandwidth UVC LED disperses as strongly as a full-range visible light LED^17^, a factor which limited image quality in previous work.

Here, we have built an intensity-based, incoherent computational QPM system that operates perfectly achromatically in the UVC regime to offer high-resolution, high-contrast and label-free quantitative microscopy. We achieved close to 200 nm resolution with over 10 times better contrast than for brightfield microscopy of identical nominal resolution. To demonstrate the versatility of our proposed system, we tested it on a particularly challenging biological sample: morphologically specialized mammalian (rat) liver sinusoidal endothelial cells (LSECs). LSECs have tiny, hard-to-visualize holes called fenestrations, which have so far never been visualized in label-free far-field optical microscopy.

## Results

To verify our assumption that UVC illumination leads to better image contrast, we assessed the effect of illumination wavelength on the achievable contrast in conventional brightfield of formaldehyde-fixed liver cells, with “contrast” measured as standard deviation normalized by image median (Fig. 1b). A detailed description is given in the Methods section. From the visible toward the UVB region, we found a slow rise in contrast with decreasing wavelength, followed by a marked jump in contrast for the UVC region. Generally, we see a high correlation between our measured contrast values, performed on liver cells, and liver tissue absorbance measurements found in the literature^13^. Note that increasingly stronger absorption at shorter wavelengths is a trend present in most tissue types^10–12^ and, hence, it is reasonable to assume the found correlation between absorption and contrast to be valid also for other tissue and cell types.

Therefore, UVC light provides an avenue for retrieving both the real and imaginary refractive index distribution of a sample. The real part modifies the illumination light’s wavefront while the imaginary part modulates its amplitude (see Fig. 1c). To disentangle both parts from measured intensity images, a dedicated retrieval algorithm can be used^14^.

**Fig. 2:**
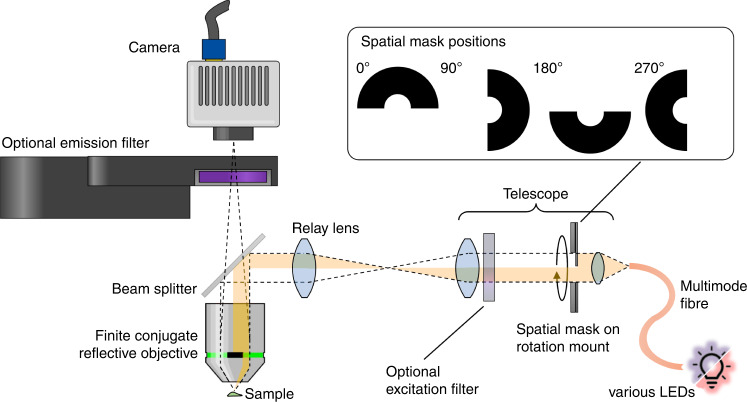
Quantitative UVC microscope illuminated using a large-core multimode fiber that collects narrowband LED light. The fiber’s end-facet is relayed to the image plane through a lens relay that contains a rotatable half-ring mask in a pupil plane of the objective (see inlay). The light reflected from the sample is recorded by a finite conjugate Cassegrain-type objective (NA = 0.65) through a beam splitter onto a UV-sensitive camera. Emission and excitation filters can optionally be inserted into the beam path for (auto-) fluorescence microscopy

Practically, however, microscopy below the UVA regime is challenging. For instance, commonly employed objectives do not transmit UVC light as their constituent lenses are made from UV-opaque materials. The limited pool of available glasses (essentially fused silica and calcium fluorite) thus renders chromatic correction in the UVC regime challenging^18^. Furthermore, glass refractive indices exhibit much stronger dispersion at short wavelengths: a narrowband UVC light source might experience as much dispersion as a broadband source spanning the whole visible range^17^. For these reasons, we opted to construct the detection arm of our microscope using a mirror-based high-NA Cassegrain-type objective in finite conjugation to avoid chromatic aberrations and minimize transmission losses. We also chose a reflection geometry to allow a double pass of the light through the sample (see Fig. 2).

To retrieve complex refractive index distributions, we opted to employ the recently developed reflection quantitative differential phase contrast (qDPC) method by Matlock et al.^14^. This inverse scattering algorithm requires a set of obliquely illuminated intensity images for phase retrieval. In our setup, we therefore included a fused silica lens relay that provides access to a conjugate pupil plane and placed a rotatable semi-circular amplitude mask there. The mask blocks half the pupil and shields the objective’s secondary mirror from on-axis illumination. This is crucial, as uncontrolled back-reflections from this mirror and associated stray light would obscure any signal-carrying light from the sample. We furthermore conjugated the output facet of a large diameter solarization-resistant multimode fiber to the image plane, which resulted in supreme field homogeneity, suppressed speckle noise, and limited the light exposure to the field of view. A schematic of the microscope is depicted in Fig. 2. For UVC illumination, we chose an LED with wavelength *λ* = 275 nm. The narrow 10 nm bandwidth minimizes chromatic effects (which are more tolerable in the illumination path) over the fused silica lenses and ensures a better-defined operating wavelength for the retrieval algorithm.

The theoretical resolution of this arrangement is ${\Delta}x = \frac{\lambda }{{2NA}} \approx 212\,{{{\mathrm{nm}}}}$. We expected a slightly worse resolution in practice due to the limited aberration correction capabilities of the objective. Put to the test on a high-resolution USAF target with up to 3300 line pairs per millimeter, our microscope resolved all available line pairs. This renders the resolving power at least 303 nm (see Supplementary Fig. 1). To gauge resolution beyond this, and as an orthogonal measurement, we used phase decorrelation^19^ as a metric on HeLa cells and LSECs. Without any further processing, we measured a resolution of ~240 nm. After qDPC processing, which incorporates deconvolution, we achieved down to ~215 nm resolution and thus a performance close to the nominal diffraction limit of our system.

**Fig. 3:**
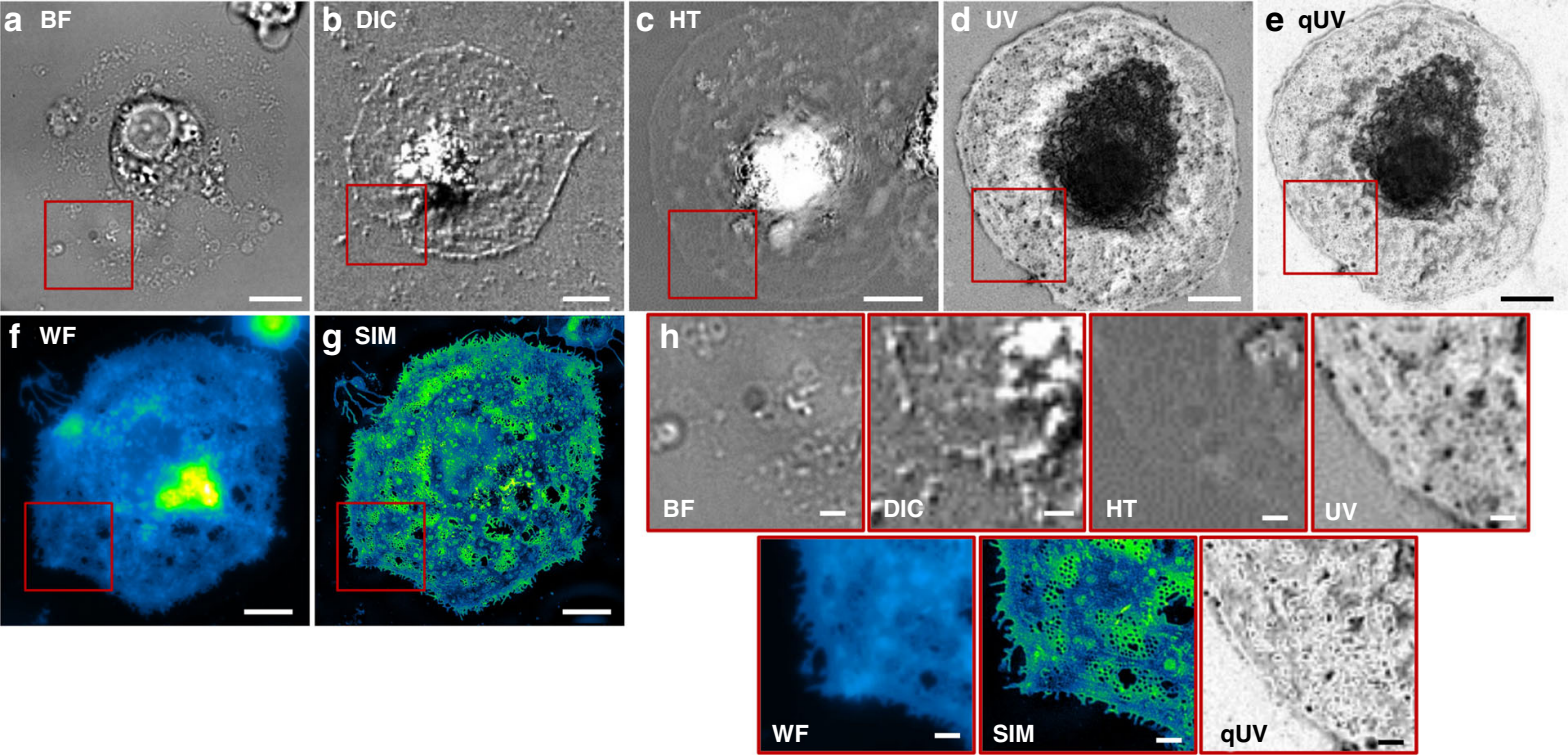
LSEC micrographs. **a**–**c** Label-free imaging of fixed LSECs using visible light brightfield (BF), differential interference contrast (DIC), quantitative holotomography (HT), and using our UV microscope (**d**, **e**) showing either a (**d**) raw UV intensity image or (**e**) quantitative UV extinction (qUV). (**f**, **g**) Fluorescence imaging of membrane-labeled LSECs using widefield (WF) and superresolution structured illumination microscopy (SIM). **h** 2.5x enlarged views of similar regions. The scale bars are 5 µm in (**a**–**g**) and 1 µm in (**i**). Label-free images were computationally adjusted with unsharp masking for enhanced visibility of the sieve plate. Enlarged views of panels d and e are shown in Supplementary Fig. S4

Next, we determined the achievable contrast of our UV microscope on LSECs. LSECs are extremely thin fluid-filtration cells that contain (sub-)diffraction limit-sized fenestrations (50–300 nm), with 5–100 fenestrations clustering together as sieve plates^20^. Fenestrations function like holes in a sieve and, due to their size, are challenging or even impossible to visualize using conventional light microscopy. This is qualitatively shown in Fig. 3a, where an LSEC, recorded in brightfield microscopy, is displayed. The figure further compares DIC microscopy (using the green part of the visible spectrum for illumination) as a non-quantitative high-contrast method, a commercial holotomography system (HT) as a quantitative method with 520 nm laser illumination, as well as our UVC microscope. Both a UVC intensity image as well as a qDPC-processed quantitative UV (qUV) image are used for the comparison. In addition, we used label-based fluorescence microscopy and nanoscopy with stained cell membranes to provide a reference for the expected structures of our cells’ sieve plates. Conventional widefield microscopy resolves sieve plates and the superresolution technique structured illumination microscopy^21^ can resolve fenestrations^22^. The theoretical resolution supported by different label-free optical microscopy methods shown in Fig. 3a–e are comparable (BF: 230 nm, DIC: 330 nm; HT: 330 nm/180 nm for raw images and processed according to the manufacturer’s specification; qUV: 215 nm). It is evident that the superior contrast supported by qUV enables the visualization of individual fenestration close to the diffraction limit (215 nm), contrary to other label-free methods. This highlights the core problem associated with label-free imaging of thin and nanoscale biological specimens that do not scatter significantly. Such structures, despite being well within the resolution limit, are not imaged due to poor contrast. This makes exploitation of extinction coefficients an attractive route to obtain superior contrast from thin and nanoscale biological samples due to enhanced absorption at UVC wavelengths.

Quantification of the achievable sieve plate contrast shows that, without any processing, UVC microscopy provides almost doubled contrast over brightfield microscopy, performing on par with DIC. When processed using the qDPC algorithm, we found an additional improvement over DIC of almost 7 times. Interestingly, we measured much lower sieve plate contrast in holotomography compared to the other modalities. Table 1 provides an overview of the contrast measurements.

**Table 1:** Comparison of LSEC sieve plate contrast between various label-free methods

	Contrast on sieve plate [σ/M]	Improvement with qUV	Contrast on sieve plate [Michelson]	Improvement with qUV
Brightfield (BF)	0.048	12x	0.257	4x
DIC	0.084	7x	0.368	3x
Holotomography (HT)	0.002	293x	0.006	154x
Type-C UV (UV)	0.085	7x	0.330	3x
qDPC UV (qUV)	0.586	–	0.998	–

The metric for contrast was either the standard deviation σ of pixel values in the sieve plate region, normalized by the median value M or the approach by Michelson (peak-to-peak)^23^

**Fig. 4:**
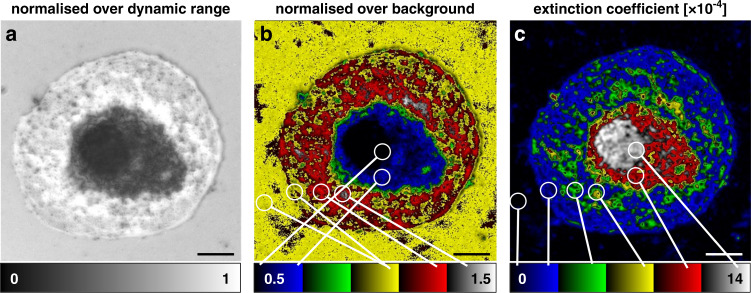
Extinction coefficient measurements of LSECs. **a** LSEC raw intensity image normalized over the dynamic range of the camera. **b** Normalization to the background of the flat-field corrected intensity image. Due to interference of illuminated and scattered light, the sieve plates appear with pixel values above both background and nucleus (yellow/blue vs red/white in the used 5-ramp colormap). **c** Extinction coefficient map of the same LSEC. After qDPC processing, the complex phase gives access to quantitative extinction coefficients. The sample now shows values linearly increasing from the non-absorbing background to the strongly absorbing nucleus. The white encircled areas in (**b**) and (**c**) show patches of various cellular compartments (background, rim, plasma membrane/sieve plates, cell body, nucleus) that exemplify the qualitativeness or quantitativeness of the two modalities, respectively. Scale bars are 5 µm

When retrieving extinction coefficients from UVC raw data, the quantitative character of qDPC processing becomes evident. Figure 4a displays an LSEC intensity image, normalized to the dynamic range of the sensor. The nucleus is dark, as expected from an image based on absorption. The thin sieve plates surrounding the nucleus, however, display pixel values *greater* than the background. When normalizing the image to the background rather than the dynamic range and applying a 5-color ramp, this back-and-forth of values becomes even clearer (Fig. 4b). We attribute this effect (seemingly smaller-than-zero absorption) to interference between the illumination and scattered field components, akin to interferometric scattering (iSCAT) microscopy^24^. While the coherence length of the employed LED is only 6.8 µm, this is sufficient to allow interference between the top and bottom of the sieve plate to occur, even when taking a double pass into account. The nucleus is much thicker and thus interference effects are reduced. In qUV, the qDPC algorithm takes interference between reflected and scattered light into account and can thus retrieve the underlying refractive indices from the DPC raw frames. As highlighted in Fig. 4c, the thus generated extinction coefficient map displays the lowest absorption in the background, highest values in the strongly absorbing nucleus and in-between values in the flat plasma membrane areas that house the sieve plates. The background has extinction coefficient values close to zero.

**Fig. 5:**
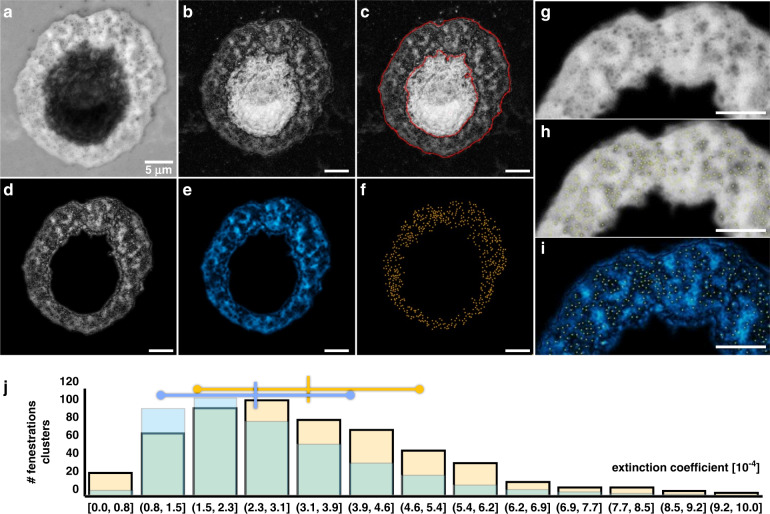
Analysis of fenestration clusters. **a** UVC LSEC image. **b** Image after qDPC processing. **c** Segmentation of the plasma membrane using a Weka classifier^25^. **d**–**f** Separation of plasma membrane (blue) from fenestration clusters (orange) using a sliding paraboloid filter. **g** Enlarged view of (**a**). **h**, **i** Enlarged views of (**a**) and (**d**) with marked fenestration clusters. **j** Histogram of detected fenestration cluster extinction coefficients from (**f**) in yellow and extinction coefficient histogram in blue of the segmented plasma membrane in (**e**). The scale bars are 5 µm

The quantitative extinction maps together with the high resolution and contrast allowed us to segment individual potential fenestration clusters from the images and determine their respective extinction coefficients. In the cell displayed in Fig. 5, we detect about 650 fenestration clusters and measure their average extinction coefficient to be (3.4 ± 1.8) × 10^–4^, higher than the extinction of the plasma membrane of (2.7 ± 1.4) × 10^–4^.

The presence of an actin skeleton around the fenestration has been reported by Mönkemöller et al.^26^ and it has been demonstrated that these nanoscale fenestrations are dynamic in nature, i.e. they close, open and merge^27^. We thus assume that the density of cytoskeletal protein is higher around the fenestrations, which leads to a higher absorption despite the missing biological material in the “holes” of the fenestrations. As most fenestrations are smaller than the diffraction limit, their rims and immediate surrounding cellular matter are likely to contribute significantly to the measured values that are presumably higher than the average extinction coefficient values found.

Apart from increasing contrast and enabling extinction coefficient measurements, deep UV illumination is capable of causing autofluorescence^28–30^, particularly by exciting intrinsically fluorescent amino acids^31^. The excitation peak of tryptophan for instance lies around 275 nm with red-shifted emission at around 350 nm. However, when measured on LSECs we detected only very small amounts of signal in the sieve plates, and only a diffuse glow from the nuclear region, which was insufficient for high-resolution images (see Supplementary Fig. S3).

## Discussion

In this work, we demonstrated the advantage of exploiting extinction coefficients for superior contrast imaging both qualitatively and quantitatively, a possibility until now not fully harnessed within microscopy. We attribute the 7-fold contrast improvement in quantitative UV microscopy over visible light microscopy to several factors. Firstly, the higher absorption coefficient acts as an intrinsic contrasting agent, while the reflective imaging geometry doubles the effective path length. Further, despite the light loss by the Cassegrain objective’s obscuration, the ring-like pupil acts as a high-pass filter, which adds to the superior image contrast of small details. Thirdly, the qDPC algorithm incorporates inverse filtering and thus performs processing steps akin to deconvolution. Finally, despite the nominally higher NA of the DIC microscope, our UV microscope provides higher resolution and can thus visualize small features better.

Without any processing, UVC microscopy provides a similar contrast improvement as DIC over brightfield illumination but, to our surprise, holotomography showed by far the lowest nominal contrast and was almost 300 times weaker than quantitative UV. Potential factors are the long depth of field of the employed holotomography system (Δ*z* = 1.3 μm), the generally weak refractive index contrast of sieve plates in the visible regime and a potentially limited sensitivity of the holotomograph’s built-in camera.

How accurate are the stated extinction coefficient measurements based on qDPC processing? Extinction coefficient measurements of background values should and do not change between samples and are generally found to be close to zero. Nevertheless, we concluded that the computationally retrieved values obtained using the qDPC algorithm must be taken as semi-quantitative, i.e., absolute values might display incorrect extinction coefficients that cannot be compared between different sample types. Our conclusion is based on several factors: firstly, biological samples generally contain intrinsically fluorescent proteins and amino acids (especially in the deep UV), whose autofluorescence signal is not accounted for in the phase retrieval algorithm. QPM imaging of fluorescently labeled biological samples is routinely performed by the community, but this effect has to our knowledge not been systematically assessed. Secondly, the Cassegrain objective’s depth of field is much larger than the imaged sample and thus underestimates the true value of the cellular material itself. This might also complicate measurements taken on the same sample type – in our LSEC experiments, some cells appeared to have a more rounded cell body and thus would fill in more volume of the objective’s depth of field. This is not possible to exclude with the presented setup and is not accounted for in the algorithm. Thirdly, the qDPC algorithm has been reported to offer limited accuracy in the presence of thicker as well as non-weak light-matter interactions^14^, which could happen for instance in the absorbing nucleus and cell body of LSECs. Many of these points could be alleviated in the future through improved algorithms or a modification of the microscope to a transmission setup geometry akin to holotomography in conjunction with an interferometric read-out.

Compared to label-based fluorescence microscopy, label-free imaging with UVC light manages to visually match the widefield performance on LSEC fenestrations, with equivalent resolution and visibility of very fine structures. The pronounced ragged rim with filopodia (spike-like projections) of the plasma membrane (visible in widefield and SIM images shown in Fig. 3f, g) can be attributed to biological variability and variations when sample cells explore their environment. Although not present in the shown LSEC in Fig. 3e, we found similar ruggedness with UVC microscopy as well (see Supplementary Fig. S3).

The achievable field of view when operating at the Nyquist limit was limited by the size of our camera chip to approximately 120 × 120 µm^2^. This is comparable to other high-resolution techniques in the visible regime and a marked jump over previous UVC methods^9,15^. As larger camera chips become available in the deep UV, we expect the limiting element to become the Cassegrain objective, which would then restrict the field of view to a 340 µm diameter according to the manufacturer. The flat illumination generation via multimode fiber is simple to scale by choosing a larger core and thus is unlikely to be an obstacle.

We also successfully demonstrated correlative high-contrast UVC and autofluorescence imaging. Due to the all-reflective detection path, high-resolution imaging without chromatic aberrations provides another layer of information from unlabeled samples. However, the long imaging times for autofluorescence signal capture (e.g., 50 s for Supplementary Fig. S3) and the phototoxicity of UV light render this setup less feasible for long-term live-cell imaging. UV radiation is highly energetic and, although not yet ionizing in the UV C-band, does provide significant stress to cells^32,33^. Although higher intensity UVC sources might provide an avenue to acquire crisp snapshots of samples before the UV light exposure triggers cellular changes, we believe that the strength of this new technique lies within superior contrast label-free imaging of fixed cells (see Supplementary Note 1 for a detailed discussion). For example, light microscopy, even in the C-band, has the crucial benefit over electron microscopy that wet samples can be used.

We believe UVC microscopy as a tool, besides being used as label-free high-resolution microscopy platform, will also find applications to quantify the extinction coefficient of biological materials, possibly as cellular fingerprints. Measurement of extinction coefficients of cells has mostly been overlooked due to the lack of a suitable tool that can probe the absorption of cells, which are, in essence, transparent in the visible regime. It is also possible to use near-IR/mid-IR wavelengths where the absorption spectrum has peaks, but images from such long wavelengths would suffer from loss of resolution and a lack of high-efficiency cameras.

Looking beyond imaging applications for animal cells, we imagine this correlative imaging approach to have a significant application potential also for the imaging of e.g., tissue sections in pathology or specimen from completely other groups of organisms like plants or fungal cells, or even bacteria and bigger viruses, which might exhibit stronger autofluorescence and may be more resilient to UVC illumination.

We presented a C-band UV microscope operating at 275 nm, which provides high-contrast high-resolution label-free microscopy. Through a series of oblique illuminations, we showed differential phase contrast (DPC) illumination in this wavelength regime and achieved a 7- to 300-fold improvement over other methods. The phase retrieval algorithm of quantitative DPC microscopy permitted us to calculate extinction coefficients within liver sinusoidal endothelial cells (LSECs). With a resolution down to 215 nm, we could resolve individual fenestrations within their sieve plates, and demonstrated the quantitative character of extinction coefficient imaging. The UVC illumination allowed us to utilize intrinsic fluorescence from proteins and amino acids as an orthogonal imaging modality. We exploited this in performing correlative label-free imaging with both autofluorescence and differential phase contrast.

## Methods

### Microscope

A diagram of the developed microscopy system is shown in Fig. 2. We chose a reflection geometry to allow a double pass of the probing light and an all-reflective detection path based on a Cassegrain-type reflection objective in finite conjugation to avoid chromatic aberrations. The UVC light was generated by a 275 nm LED (Thorlabs) with narrow bandwidth, which was spatially flattened by a large-core solarization-resistant multimode fiber. Other wavelengths were used by swapping the UVC LED with LEDs emitting in the UVB and UVA, as well as LEDs from the blue and red ends of the visible spectrum (300 nm, 340 nm, 365 nm, 625 nm, all Thorlabs). The fiber output was then collimated with a fused silica aspheric lens (Edmund Optics) and relayed onto the sample by UVC transmissive singlet lenses made of fused silica. Furthermore, the illumination path was equipped with a rotatable half-ring mask in an aperture plane to generate oblique partially coherent illumination light that is suitable to generate raw data for quantitative differential phase contrast (DPC) microscopy. The employed illumination relay deviated from a nominal 4f relay (optimized via raytracing in Optics Studio) to accommodate the finite conjugation of the detection path while still mapping the aperture mask onto the back focal plane of the objective and the fiber end face onto the image plane. The light was reflected off of a dichroic beam splitter (F38–266, AHF, Germany), which acted as a 50/50 beam splitter at 275 nm illumination. Reflected light was captured by a Cassegrain-type finite conjugate reflective objective with a nominal numerical aperture of 0.65 (5006, Beck Optronic, UK). The objective’s two mirrors were coated with aluminum to increase the overall transmittance of the objective. The upright reflection geometry permitted imaging of samples without coverslip in shallow aqueous solution of defined depth or of samples with UVC transmissive fused quartz coverslips on top. When swapping between both conditions, aberrations were minimized by adjusting the mirror spacing inside the objective with a correction collar to accommodate the change in optical properties on the sample side. When imaging through a water column instead of cover glass, a correction factor of 0.9 to the equivalent cover-glass thickness setting yielded best image quality and diffraction-limited resolution. The tube length, i.e., the distance to the camera from the objective was set to about 350 mm, which resulted in an effective magnification of 105x and was thus sufficient for Nyquist-sampling the 11μm pixel size chip of the employed UV sensitive sCMOS camera (95B Blue, Teledyne, USA).

### Contrast measurements in brightfield (Fig. 1b)

Following the method by Moulden et al.^34^, we used the standard deviation of an image as a metric for contrast. The standard deviation of intensity images was normalized to their respective median values to allow comparability between images acquired at different wavelengths and thus different illumination intensities. As a control, we also calculated contrast based on Michelson’s approach:^23^ $c_{{\rm{Michelson}}} = \frac{{I_{{{{\rm{max}}}}} \,-\, I_{{\rm{min}}}}}{{I_{{\rm{max}}} \,+\, I_{{\rm{min}}}}}$. We acquired images of matching fields of view with narrowband LED illumination at five different wavelengths covering the UVC, UVB, UVA, blue and red part of the spectrum: 275 nm, 300 nm, 340 nm, 365 nm, 625 nm. Illumination intensity and exposure time were adjusted for each image separately to utilize the camera’s dynamic range.

### Imaging procedure

#### UV and quantitative differential phase contrast (qDPC) microscopy

Four raw oblique illumination images were taken corresponding to four rotational positions of the spatial mask as shown in the inlay of Fig. 2. The illumination profile in the pupil covered a semi-ring up to the full NA of the 0.65NA Cassegrain objective. Central parts of the NA were blocked due to the objective’s obscuration. Illumination light in the pupil was furthermore found to be weaker at the highest spatial frequencies compared to intermediate ones due to the emission profile of the employed multimode fiber. For regular intensity UVC microscopy, the four obliquely illuminated intensity images were averaged without further processing.

For qDPC microscopy, we additionally acquired background images at the same four oblique illumination directions. Background images were taken on an area of the sample void of cells. Raw images could thus be flat field corrected before phase retrieval. We used a modified MATLAB reconstruction software originally developed by Matlock et al.^14^ to calculate extinction coefficient maps. Using regularization parameter τ values between 100 and 1000 proved a good range for our samples to balance between artefacts and resolution. However, as this parameter setting depends on SNR a general recommendation cannot be given. Note that a higher value for τ may reduce the nominal resolution (measured by phase decorrelation), while a too low value can cause patterned artefacts as commonly seen with Wiener filtering when using too small of a Wiener parameter (see Supplementary Fig. S5). Apart from oblique illumination raw images, the software requires the pupil function, an estimate of the depth of field, as well as the illumination parameters (in our case purely the directions of oblique illuminations) as inputs.

##### Pupil function

To determine the pupil function, we changed the semi-circular amplitude mask of our microscope with a mask containing an off-center pinhole. The optical transfer function of images acquired with this arrangement consist of the sample’s spectrum multiplied by two decentered copies of the pupil function (see Supplementary Fig. 1a). To obtain a noise-free estimate of the coherent transfer function, we located the decentered pupil functions using an object detection neural network originally developed for Fourier ptychography reconstruction parameter estimation^35^, averaged the thus extracted pupil functions of multiple images, and binarized them.

##### Depth of field

The qDPC algorithm calculates extinction coefficients based on the objective’s depth of field. A Cassegrain objective’s depth of field is longer compared to a conventional objective of equal numerical aperture as parts of the generalized pupil are effectively blocked by the secondary mirror. In our case, spatial frequencies below an equivalent 0.27 NA are excluded from the pupil. To calculate the correct depth of field, we thus use a *truncated* Ewald sphere as geometric model of the generalized pupil, and find the depth of field to be ${\Delta}z = \frac{{\lambda _0}}{n}\left( {\sqrt {1 - \frac{{NA_{obs}^2}}{{n^2}}} - \sqrt {1 - \frac{{NA^2}}{{n^2}}} } \right)^{ - 1} = 1.355\,\mu {{{\mathrm{m}}}}$. This is longer than the expected thickness of LSEC sieve plates (0.1–0.2 µm) and filopodia (0.1–0.3 µm)^22^, so our calculated extinction coefficients are likely to underestimate the true values.

##### Illumination direction

The main illumination parameter used within the reconstruction is the directionality of oblique illuminations. As the amplitude mask direction is generally not aligned with the rotation mount’s position sensor, we sampled oblique illumination angles at fine steps and chose those rotations that coincided with X and Y directions. To determine the respective illumination directions, we developed software in MATLAB to determine the illumination direction from intensity images. The algorithm uses the fact that a semi-circular aperture results in an anisotropic optical transfer function. An image with small features (e.g., polystyrene beads or bits of scattering debris) thus yields a spectrum dominated by the transfer function shape. Radial projections of the spectrum show a maximum value in the direction orthogonal to the average illumination direction. We find that a naïve implementation of this approach does not provide an accurate estimation of imperfect raw images due to processing artifacts, but filtering and pre-processing steps alleviate this issue. In detail, our software crops an input image with a large circular mask with a smooth edge (Gaussian blur with sigma = 20 pixels) and extracts its periodic component using periodic-smooth decomposition. Both steps reduce edge effects. The periodic part is Fourier transformed and the logarithmic magnitude of the spectrum is cropped with another smooth mask, to exclude spatial frequencies outside the nominal support and enhance high spatial frequencies. The result is Radon transformed and the projection values through the center for each angle are filtered with a 3-pixel wide median filter for increased robustness. The maximum value of this calculation is orthogonal to the illumination direction. Examples of simulated and real data are shown in Supplementary Fig. S2.

#### Widefield (WF) and structured illumination microscopy (SIM) of LSECs (Fig. 3f–h)

For widefield and SIM, LSECs were labeled with CellMask Green (1:1000 in serum-free RPMI) in a culture dish without washing. The labeled cells were imaged using a commercial super-resolving SIM (DeltaVision/OMXv4.0 BLAZE, GE Healthcare) with a 60×1.42NA oil-immersion objective (Olympus). 3D-SIM image stacks of 1 μm were acquired with a z-distance of 125 nm and with 15 raw images per plane (five phases, three angles). Raw datasets were computationally reconstructed using “SoftWoRx” software (GE Healthcare). Figure 3h shows a single z slice.

Widefield images were acquired on the same microscope with identical sample and imaging parameters.

#### Differential interference contrast microscopy (DIC) (Fig. 3b)

DIC imaging was done using a Deltavision Elite Microscope (GE Healthcare) equipped with an sCMOS camera (PCO Edge 5.5 with a 6.5 µm pixel size), 0.55 NA long working distance condenser, and 20×0.75 NA air objective (Olympus). Both a diffuser and green filter (GE Healthcare) were used with the LED illumination.

#### Holotomography with Nanolive (NL) (Fig. 3c)

Holotomographic images were acquired using a 3D Cell Explorer-fluo (CX-F, Nanolive), which uses a Sony CMOS IMX174 camera sensor with 5.86 µm pixel size. The system is equipped with a 60×0.8NA air objective and uses a low power (0.2 mW/mm2), 520 nm laser for imaging. Images were automatically reconstructed using the manufacturer’s provided software, “Steve”. Figure 3c shows a single z slice from the reconstructed volume.

### Fenestration analysis (Fig. 5)

UVC differential phase contrast images were processed into qUV images as described above. Using the Weka (Waikato Environment for Knowledge Analysis) segmentation plugin^25^ in Fiji^36^, we segmented the plasma membrane from the background and the nucleus. Fenestrations were extracted from the plasma membranes by applying a 10-pixel sliding paraboloid background removal filter in Fiji. Fenestrations were than detected using a maximum filter with 0.00015 prominence.

### Sample preparation

#### Animals and ethics statements

Sprague Dawley male rats (Animal Resource Centre, Murdoch, Western Australia) were group-housed (2–3 rats/cage) and kept under controlled environmental conditions (21 °C ± 1°, relative humidity 55% ± 5% and 12 h light/12 h dark cycle) with standard chow *ad libitum* (Glen Forrest, Western Australia) feeding. The experimental protocols and animal handling were approved by the ethics committee of the Sydney Local Health District Animal Welfare Committee (Approval 2017/012 A). All experiments were performed in accordance with relevant approved guidelines and regulations. All animals were euthanized and died from exsanguination while in deep surgical anesthesia during the liver perfusion procedure (for anesthesia protocol see section “Rat LSEC isolation and cell culture”).

#### Material for LSEC preparation

Human fibronectin was purified from human plasma by affinity chromatography on Gelatin Sepharose 4B as described by the manufacturer (GE Healthcare, Uppsala, Sweden). Serum-free Roswell Park Memorial Institute (RPMI-1640) cell culture medium (supplemented with 20 mM sodium bicarbonate, 0.006% penicillin, and 0.01% streptomycin) and monensin were from Sigma-Aldrich (Sydney, AU/Oslo, Norway). Culture dishes of 35 mm diameter with 20 mm #1.5 glass bottom were from VWR International (Rador, PA, USA). CellMask™ Green was from Fisher Scientific (Cat No. C37608; Oslo, Norway).

#### Rat LSEC isolation and cell culture

The rats (body weight 300–400 g) were anesthetized with a mixture of 10 mg/kg xylazine (Bayer Health Care, CA, USA) and 100 mg/kg ketamine (Ketalar, Pfizer, NY, USA) in saline. LSECs were isolated and purified as described in Smedsrod, et al.^37^, cryopreserved storage for LSECs followed with freezing and thawing were detailed in Mönkemöller et al.^38^ The cells were plated (0.2 × 10^6^ cells/cm^2^) in serum-free RPMI-1640 on 0.2 mg/mL human fibronectin-coated 0.2 mm thick fused quartz coverslips (AdValue technology, USA), unattached cells were gently washed away, and LSEC cultures were continued with incubating for 1–2 h to reach the desired confluency. Fibronectin coating was performed with just enough volume to completely cover the surface area. After 10 min of incubation at room temperature, the redundant fibronectin solution was rinsed with PBS and cells were then seeded on UVC transmissive fused quartz coverslips (200 µm thickness, AdValue Technology, USA). LSECs were fixed with 4% formaldehyde (FA) in phosphate-buffered saline (PBS) and 0.02 M sucrose (Sigma-Aldrich), pH 7.2 for 15 min, and stored in PBS until visualization.

#### Autofluorescence imaging

The exposure time for autofluorescence imaging was 50 s with an intensity of approximately 0.2 mW of 275 nm illumination light. The illumination intensity is reduced from the nominal emission of the 80 mW LED by incorporating losses due to coupling into the multimode fiber (550 µm diameter vs 2x2mm emitting LED surface), the pupil mask with obscuration half-sided block, as well as the losses in the 50:50 beam splitter: $80\,{{{\mathrm{mW}}}} \times 0.5 \times 0.5 \times \left( {\frac{{0.27^2}}{{0.65^2}}} \right) \times \frac{{\left( {\pi \times \left( {\frac{{0.55\,{{{\mathrm{mm}}}}}}{2}} \right)^2} \right)}}{{2\,{{{\mathrm{mm}}}} \,\times\, 2\,{{{\mathrm{mm}}}}}} \approx 0.2\,{{{\mathrm{mW}}}}$.

## Supplementary information

supplementary information
