# Supplementary material for: Label-free superior contrast with c-band ultra-violet extinction microscopy
Source: Light Sci Appl. 2023 Mar 3;12:56. doi: 10.1038/s41377-023-01105-6 (PMC9981877; doi:10.1038/s41377-023-01105-6)
Supplement: Supplementary file 1 — supplementary information [file 41377_2023_1105_MOESM1_ESM.docx]

# Supplementary information: Label-free superior contrast with c-band ultra-violet extinction microscopy

Florian Ströhl 1*, Deanna L. Wolfson 1, Ida S. Opstad 1, Daniel H. Hansen 1, Hong Mao 1, Balpreet S. Ahluwalia 1,2

1 Department of Physics and Technology, UiT The Arctic University of Norway, Tromsø, Norway

2 Department of Clinical Science, Intervention and Technology, Karolinska Institute, Stockholm, Sweden

* florian.strohl@uit.no

**Supplementary Note 1: utility of UVC microscopy for label-free bio-imaging**

UV radiation is well-known to be damaging to cells and that is indeed a common application of such radiation in the context of surface and water sterilization. Hence, we do not attempt to prove live-cell or even time-lapse imaging but instead demonstrate the use of UVC light for fixed-cell imaging. It is, however, possible to perform single-shot live-cell imaging, albeit we do not show this in the manuscript. It could also theoretically be used to assess the impact of UVC light on biological samples over time. Note that UVC microscopy works with fixed wet samples, giving it a crucial advantage over electron microscopy which is limited to fixed dry samples and operates only in vacuum. Recently [1], it has been meticulously demonstrated that the drying process of LSECs (a necessary preparation step for electron microscopy imaging) results in larger-sized fenestrations as compared to wet fixed samples using fluorescence-based super-resolution optical microscopy methods. We foresee that our proposed method will find usage primarily for fixed cell bio-imaging applications where it would out-perform existing far-field optical methods such as differential interference contrast (DIC) microscopy, holotomography, quantitative phase microscopy (QPM), or gradient light interference microscopy (GLIM) with superior contrast in a label-free mode. Note that these methods, however, can be applied to long-term live-cell imaging unlike UVC microscopy.


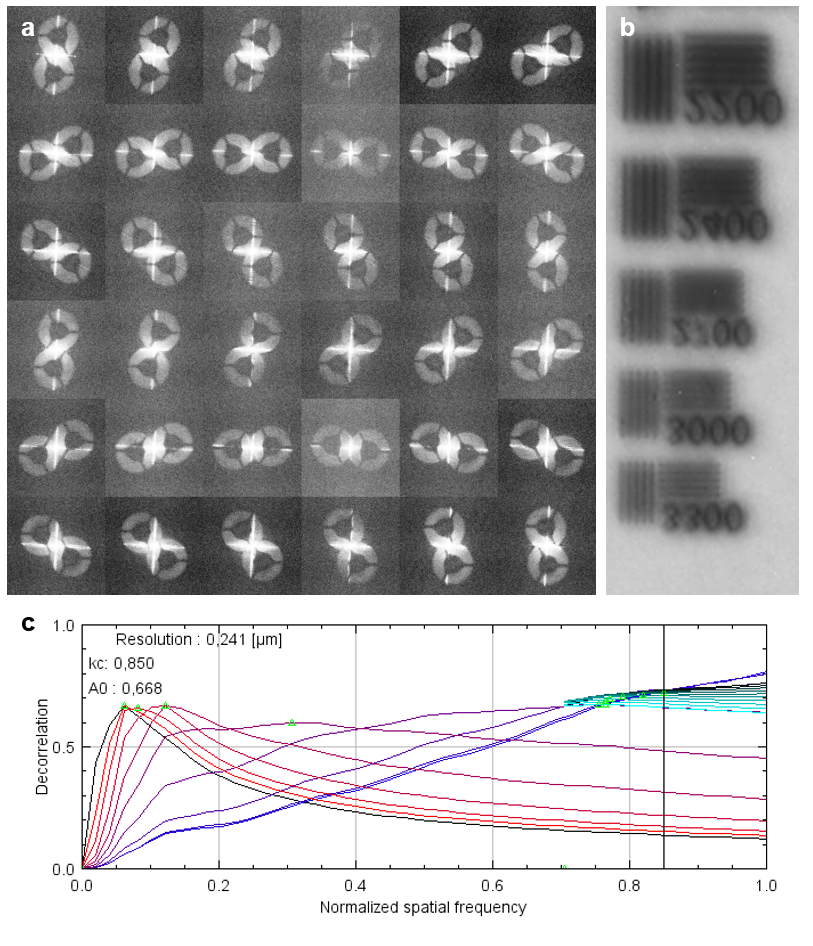


*Supplementary Figure S1: (a) Fourier transforms of images taken with a Cassegrain objective under partially coherent illumination using a mask with an off-axis pinhole in a pupil plane. Rotation of the mask results in image spectra that depict the pupil function shifted to the illumination wave vectors spatial frequency coordinate and its conjugate. Averaging of many such images provides a high-fidelity estimate of the objective’s pupil function. (b) UVC micrograph of a USAF resolution target with up to 3300 line-pairs per mm. (c) Image resolution on the USAF target as estimated by phase decorrelation analysis [2].*


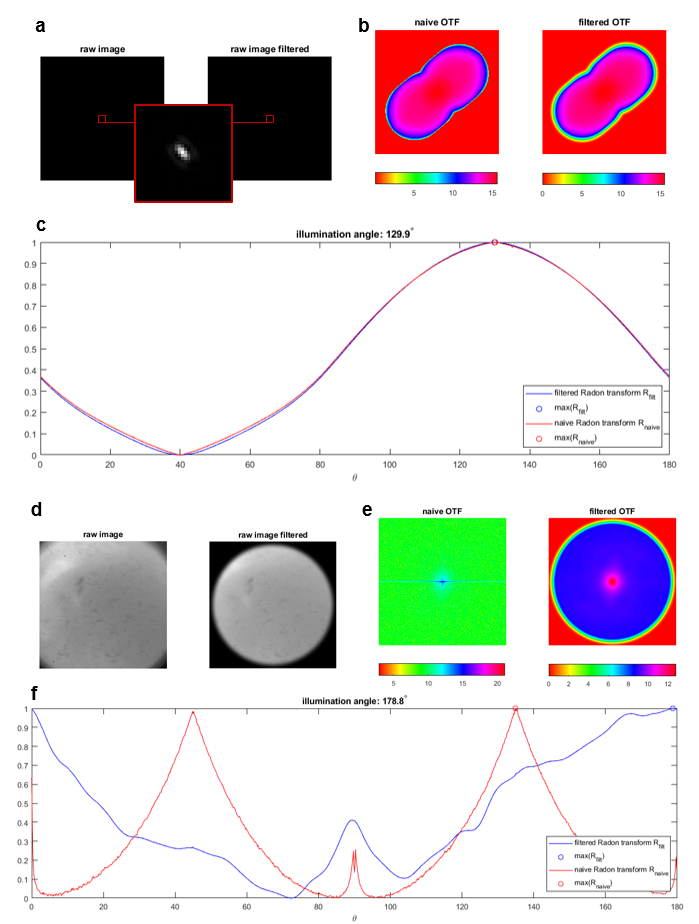


*Supplementary Figure S2: Parameter estimation software to determine the direction of the DPC illumination. (a-c) shows the performance of the software on a high-contrast, isolated scatterer in a synthetic sample. Both naïve (red line) and filtered (blue line) processing provide a good estimate of the ground truth illumination angle of 130°. (d-f) On a real sample, the weaker contrast and lower signal-to-noise ratio renders the naïve approach unusable, while filtering informs on the illumination direction (peak of the graph). In this example, the illumination mask was aligned with the x-axis, thus the peak is close to 0 or 180 respectively.*


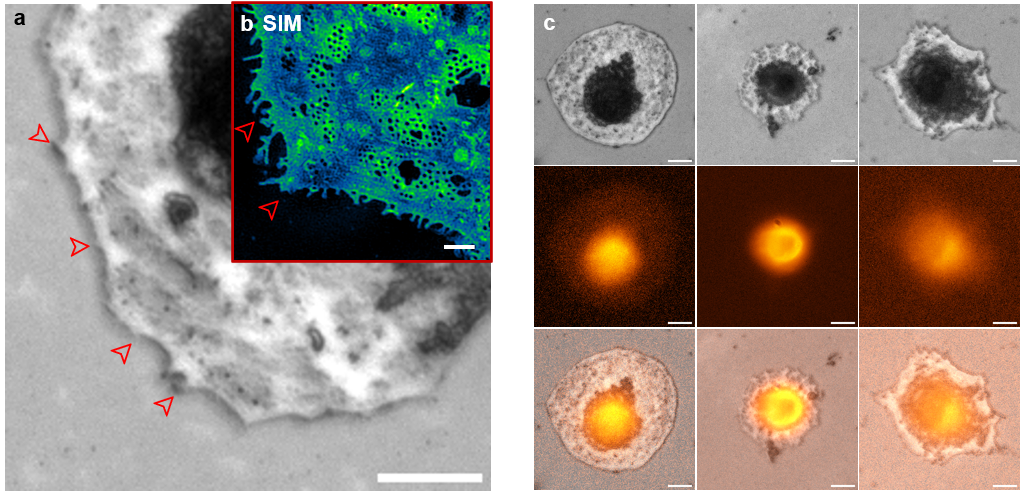


*Supplementary Figure S3: (a) LSEC UV image showing a ragged rim with filopodia (spike-like projections of the plasma membrane, red arrows), which allows the cell to explore its environment. Not all cells were found to display this feature. (b) The same ragged feature highlighted in a SIM superresolution image of a different LSEC. (c) Various LSECs imaged using UVC (upper row), correlative autofluorescence using 275 nm excitation and 357/44 nm emission (middle row), and an overlay of both modalities (bottom row). Scale bars are 5 µm in (a) and (c) and 1 µm in (b).*

*
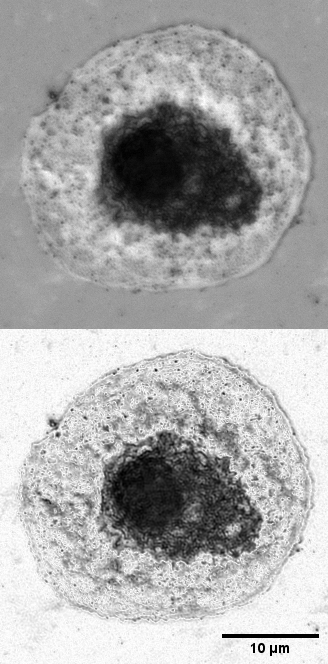
*

**b**

**a**

*Supplementary Figure S4: Larger views of (a) the UV and (b) the qUV image of an LSEC, displayed as panels (d) and (e) in Figure 3 of the main manuscript.*


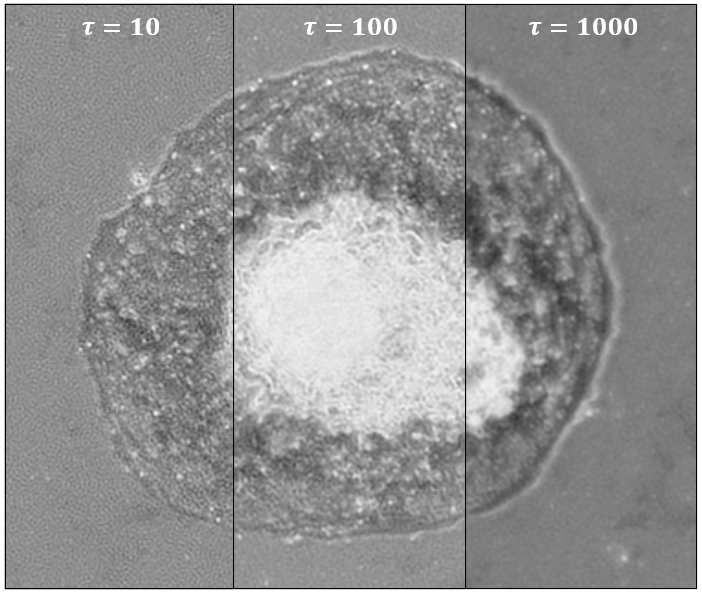


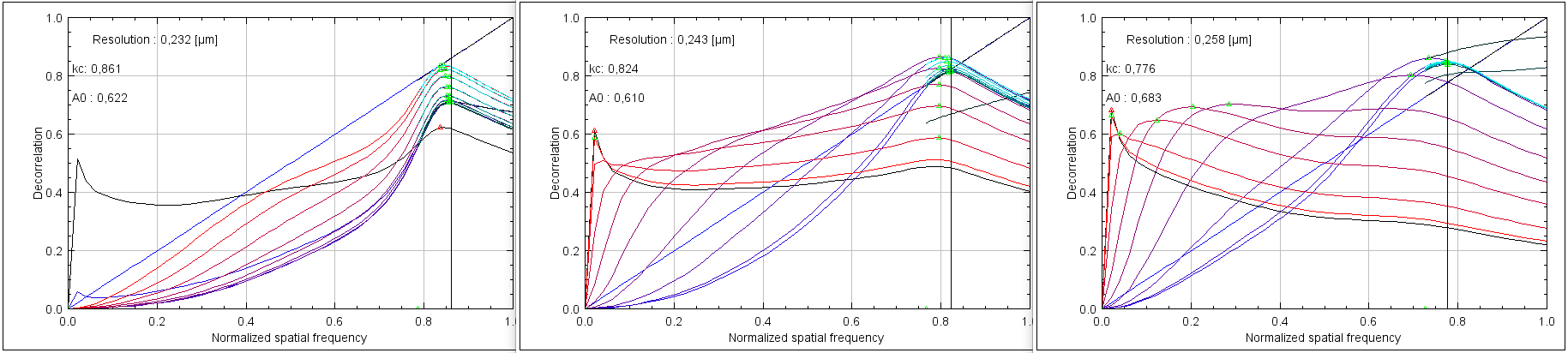


*Supplementary Figure S5: Impact of the regularization parameter τ on image quality and resolution. The generation of qDPC images follows the approach by Matlock et al. [3], incorporating a closed-form solution of Tikhonov deconvolution, and is therefore related to the generalised Wiener filter known from structured illumination microscopy (SIM). The respective regularisation (Wiener) parameters in both cases reflect the local signal to noise conditions. For a fixed parameter as is normally the case, these might be off in certain parts of the image as the local signal to noise conditions may be varied and therefore cause artifacts if the chosen value is too low. Similarly, the illumination structure in the pupil is used as an input for qDPC, which is modelled as a half-ring of the pupil with obscuration. A potential inhomogeneous light distribution in the pupil is hence not accounted for and might lead to patterned artefacts if deconvolution is too aggressive. This is shown in the upper row, where a regularisation parameter of τ = 10 results in “herringbone” pattern in the background. We have experimented with different settings for the regularization parameter and found a range of τ = 100-1000 to be good values for our samples. Changing the regularization parameter also influences the measured resolution as shown by phase decorrelation analysis [2] in the lower half of the figure. We find a higher resolution of 232 nm with a τ = 10 at the expense of more herringbone artifacts, whereas a conservative τ = 1000 results in minimal artifacts, yet at a resolution drop down to 258 nm.*

**References**

[1] Szafranska, Karolina, et al. "From fixed-dried to wet-fixed to live–comparative super-resolution microscopy of liver sinusoidal endothelial cell fenestrations." *Nanophotonics* 11.10 (2022): 2253-2270.

[2] Descloux, A., Kristin Stefanie Grußmayer, and Aleksandra Radenovic. "Parameter-free image resolution estimation based on decorrelation analysis." *Nature methods* 16.9 (2019): 918-924.

[3] Matlock, Alex, et al. "Inverse scattering for reflection intensity phase microscopy." *Biomedical optics express* 11.2 (2020): 911-926.
